# Is a larger patient benefit always better in healthcare priority setting?

**DOI:** 10.1007/s11019-024-10208-9

**Published:** 2024-06-01

**Authors:** Lars Sandman, Jan Liliemark, Erik Gustavsson, Martin Henriksson

**Affiliations:** 1 Centre for Assessment of Medical Technology, Department of Health, Medicine and Caring Sciences, 58183 Linköping, Sweden; 2 Division of Philosophy and Applied Ethics, Department of Culture and Society and Department of Health, Medicine and Caring Sciences, National Centre for Priorities in Health, Linköping University, Linköping, Sweden

**Keywords:** Healthcare priority setting, Patient benefit, Distributive justice, Prioritarianism, Egalitarianism, Sufficientarianism

## Abstract

When considering the introduction of a new intervention in a budget constrained healthcare system, priority setting based on fair principles is fundamental. In many jurisdictions, a multi-criteria approach with several different considerations is employed, including severity and cost-effectiveness. Such multi-criteria approaches raise questions about how to balance different considerations against each other, and how to understand the logical or normative relations between them. For example, some jurisdictions make explicit reference to a large patient benefit as such a consideration. However, since patient benefit is part of a cost-effectiveness assessment it is not clear how to balance considerations of greater patient benefit against considerations of severity and cost-effectiveness. The aim of this paper is to explore the role of a large patient benefit as an independent criterion for priority setting in a healthcare system also considering severity and cost-effectiveness. By taking the opportunity cost of new interventions (i.e., the health forgone in patients already receiving treatment) into account, we argue that patient benefit has a complex relationship to priority setting. More specifically, it cannot be reasonably concluded that large patient benefits should be given priority if severity, cost-effectiveness, and opportunity costs are held constant. Since we cannot find general support for taking patient benefit into account as an independent criterion from any of the most discussed theories about distributive justice: utilitarianism, prioritarianism, telic egalitarianism and sufficientarianism, it is reasonable to avoid doing so. Hence, given the complexity of the role of patient benefit, we conclude that in priority practice, a large patient benefit should *not* be considered as an independent criterion, on top of considerations of severity and cost-effectiveness.

## Introduction and aim

Consider the following situation:

Example 1: Alison and Bernadette are suffering from conditions, equally and highly severe. There are two interventions, A and B, that would benefit them. Intervention A has a large patient benefit and Alison would only have minor symptoms left if treated. Intervention B has a minor patient benefit, and Bernadette would still suffer from a condition causing a substantially impaired health level after being treated with B. If there were only resources to fund one of the interventions, which would you choose?

It is difficult to find any reasons to prioritize or choose B before A here. In this abstract situation, all else being equal, it may seem obvious that we should choose the greater patient benefit before the smaller.

Consider instead the following, more real-life, priority setting situation:

Example 2: A reimbursement board is considering whether to introduce two new pharmaceuticals, C and D, targeting equally and highly severe conditions. C and D have the same cost-effectiveness ratio, on the borderline of being viewed cost-effective given the severity of the conditions – hence it is not obvious they should be reimbursed in the healthcare system. C has a large patient benefit, whilst D has a more marginal, but still relevant, patient benefit. Should this difference in patient benefit make a difference to whether C and D are reimbursed or not?^1^

In a resource constrained healthcare system priority decisions are not only theoretical considerations but also practical ethical challenges. In distinction to example 1 (which is seldom a real priority choice since resources are not often constrained in such a way), example 2 represent a real ethical challenge, experienced by all of the authors, giving rise to the question of which role patient benefit should play in reimbursement or priority decisions. In example 2, we might have a similar intuition as in example 1, that the difference in patient benefit should make a difference to the reimbursement decision. Our aim in this article is to explore if, and if so how, patient benefit *as an independent criterion* should be taken into account in priority setting decisions given a *mid-level approach* to priority setting.

## Defining the problem and setting the scene

In guidelines for priority setting, sometimes fortified in healthcare jurisdictions, we will generally find mid-level approaches using multi-criteria frameworks or principles. These frameworks might contain traces from different more abstract distributive justice theories, but there are no examples where such priority setting is guided by a single or unified abstract theory. Such frameworks might contain different sets of criteria; treat these criteria as on par with each other—calling for a qualitative or quantitative weighing or identify rational or logical relationships between these criteria; have different legal status in terms of being mandatory or suggestions developed within the academic field etc. In this article, our objective is to provide guidance for such mid-level approaches by accepting the approach by Baltussen & Niessen 2016) according to which we argue the need to explore logical or rational relationships between different criteria in multi-criteria approaches. It might be impossible to find a set of consistent principles for priority setting, possible to apply without uncertainties for every priority setting situation within a complex healthcare system with complex responses to treatments and interventions. However, in using normative analysis, we can expose ideas of what should matter to critical inquiry and show that the role we thought they had, is unwarranted. Hence, even if abstract distributive justice theories cannot resolve these mid-level problems per se, we will argue that if a specific relationship between criteria or use of a criterion gets unanimous support, regardless of theory—it will speak strongly in favour of this relationship or use, and *mutatis mutandis* for when they speak against.

In this article we will focus on the relationship between patient benefit^2^ and other priority setting criteria such as severity and cost-effectiveness. These are criteria we find in different combinations in most multi-criteria frameworks for priority setting. In some frameworks, we find both cost-effectiveness and patient benefit as independent criteria (see for example Baltussen & Niessen 2016, the operationalization of the Swedish legal framework Prop. 1996/97:60, and in other frameworks costs and patient benefit as independent criteria (se for example Goetghebeur & Cellier 2018 and the Norwegian legal framework NOU 2014). Even in the framework adapted by NICE for England and Wales, which is largely focused on cost-effectiveness, we find references to the size of patient benefit when it concerns so called highly specialized technologies (i.e. basically orphan drugs) for accepting a radically higher cost-effectiveness threshold (NICE 2017). When cost-effectiveness and patient benefit are both considered, it is implied that the larger the patient benefit, the higher the priority. However, except for some references to the risk of double counting (see for example Goetghebeur & Cellier 2018) if taking both patient benefit and cost-effectiveness into account, we find no more in-depth analysis of this relationship.

Since cost-effectiveness is a criterion used in practice in most of the practical applications of the frameworks above, we will assume this as an established criterion also in our discussion. Hence, the more specific thesis we are analyzing in this article is:

- Ceteris paribus, a greater patient benefit of an intervention should result in a higher priority for that intervention.

In addressing a real-life priority challenge, we will make some further assumptions to set the scene. The priority situation analysed when deciding whether a new intervention should be introduced (like in the second example) assumes that patients are already treated within the system. Accordingly, introducing a new intervention means that the currently constrained resources must be redistributed. Hence, we assume that it is not possible to allocate additional resources to the healthcare system or increase the productivity of the system to free the needed resources. This implies that there is an opportunity cost, in terms of health foregone, for any priority decision made in the system. Both in order to simplify the analysis of redistribution, but also to reflect a central consideration of equity, we will allow severity of disease condition to play a central role (something it does in many of the above referred frameworks and jurisdictions). Hence, we will assume that the considerations of cost-effectiveness and patient benefit should be balanced against equity considerations. Equity concerns can be represented by accepting different cost-effectiveness thresholds for different severity levels (Siverskog 2021). When these cost-effectiveness thresholds are higher than the marginal productivity of the healthcare system (Claxton 2015, Ednay et al. 2018, Vallejo-Torres et al. 2018, Sandman & Liliemark 2018), we are willing to reduce the efficiency of the system to achieve a more equitable system.^3^

In the analysis, we will assume that the interventions discussed are clinically (and morally) relevant in the sense that there are no benefits that are so small that they are trivial and therefore should not be taken into account. Likewise, on the other end of the benefit size spectrum, we set aside benefits that could have a substantially different qualitative dimension from other benefits to the patient, e.g., cure.

Our analytical approach is to start with rather abstract examples and try to introduce more realistic features as we move along. In the examples, however, we will use individuals (for simplicity) but these individuals may also represent patient groups and we will indicate when (and if) the size of these groups are relevant to consider.

## Our introductory abstract example revisited

In our introductory example 1, we concluded that we had reason to prefer the greater patient benefit over the smaller benefit. All else being equal, greater patient benefit seems better than smaller such benefits. Consider next the introductory case from the perspective of theories of distributive justice. Let us briefly spell out three such theories that we shall employ in the subsequent analysis.

*Utilitarianism* consists of two components: a claim about what is good and a claim about what should be done with that good. Whereas the former component is defined in terms of utility, the latter prescribes maximization. Accordingly, a utilitarian principle says that the net sum of utility should be maximized (see e.g., Singer 1993; Tännsjö 1998).

*Prioritarianism* is like utilitarianism in the sense that it also says that the good should be maximized. However, it involves an additional thesis, namely that it ascribes more importance to benefits that accrue to the worse off. Accordingly, it matters more to benefit people the worse off these people are (see e.g., Parfit 1997; 2012).

Telic *egalitarianism* is a pluralistic egalitarian theory that consists of one claim about value and one claim about equality. The value claim is that more benefits is better than less benefits. The claim about equality ascribes final value to equality. That is, according to telic egalitarianism, it is bad in itself if some people are worse off than other people (see e.g., Temkin 1993). Exactly how the relationship between these two aspects of egalitarianism should be spelled out is a matter of dispute (see e.g., Temkin 1993).

To prioritize A over B in the introductory example is supported by both utilitarianism and prioritarianism. Telic egalitarianism would acknowledge that prioritizing A before B is at least better in one respect (even if equality aspects might counter that argument for priority of A over B). However, note that on a strict egalitarian theory, according to which equality is the only important value, status quo, i.e., not providing treatment to either A or B would be better – since then they remain equally situated. That strict egalitarianism recommends such a course of action, i.e., levelling down, is a standard objection against this theory (Parfit 1997), and therefore a theory about distributive justice that have very few (if any) contemporary adherents.

On the face of it, three dominating theories about distributive justice seems to imply that A should be prioritized over B. If this is true, it would seem to provide strong support for the initial intuition. In the following, we will therefore explore if the same holds under other, more realistic, conditions. In order to do so, we will have to expand the original example.

## A more realistic example and introducing cost-effectiveness

In the following we shall change our examples on the basis of the assumptions introduced above. First, that there is need for redistribution, and second, that cost-effectiveness and severity are already employed as priority-setting criteria.

Example 3: Alison and Bernadette are suffering from conditions, equally and highly severe. There are two different interventions, A and B, that would benefit them. Intervention A has a large patient benefit and Alison would only have minor symptoms left if treated. Intervention B has a minor patient benefit, and Bernadette would still suffer from a condition causing a substantially impaired health level after being treated with B. However, to treat Alison or Bernadette, you need to redistribute resources from Cecil and hence withdraw^4^* intervention C, which he has access to. How to prioritize between A, B and C in this situation?*

Let´s assume that the resources needed to use A, B and C are the same. Accordingly, we could express the examples in terms of cost-effectiveness instead of in terms of the size of patient benefit. In example 3, this would imply that A is more cost-effective than B. Let us further assume that it is ethically warranted to redistribute resources from Cecil to Alison and Bernadette, motivated by Cecil having a less severe condition and C having a lower patient benefit than B. This implies that in terms of cost-effectiveness: A < B < C—and in terms of severity without any redistribution of resources: A = B > C. Hence, if we have information about severity and cost-effectiveness it is justified to choose A before B and enable this by redistributing resources from Cecil—and this would be supported by our three different distributive theories (at least on some accounts of the telic egalitarian theory). Explicit information about the size of patient benefit is, on top of being fed into the incremental cost-effectiveness ratio, thus redundant to this decision.

Could there still be circumstances when the size of the patient benefit will be relevant to consider? One way to test this is to hold severity and cost-effectiveness equal, and explore whether the larger patient benefit should be prioritised following our hypothesis? This is what we are going to explore in the rest of this article.^5^

## Keeping severity and cost-effectiveness constant—varying patient benefit

In order to keep severity and cost-effectiveness constant and still be able to vary the size of the patient benefit, we will have to accept different costs (and opportunity costs) of the interventions and different sizes of patient groups. To illustrate this, let us quantify the severity in terms of health level, patient benefit, and cost in the following way:

- The health level can vary between 0 and 1, where 0 signifies the worst possible condition and 1 signifies a condition with no severity. So, a health level of 0.1 means a higher severity than a health level of 0.8 etc.
- Patient benefit can vary between 0 and 1, where 0 signifies no patient benefit and 1 the largest possible patient benefit.

The costs of interventions are incurred in monetary terms, but can be converted to health opportunity costs, i.e., the patient benefits the monetary resources could have created elsewhere in the constrained healthcare system.

Modifying example 3 accordingly we can consider example 4:

Example 4: Alison and Bernadette are suffering from conditions, both having a health level of 0.1, and hence a very high severity. There are two interventions, A and B, that would benefit them. Intervention A has a patient benefit of 0.8 and Alison would only have minor symptoms left if treated. Intervention B has a patient benefit of 0.4, and Bernadette would still suffer from a condition with a health level of 0.5 (and hence moderate severity) if treated. To treat Alison or Bernadette, you need to redistribute resources from Cecil and hence withdraw intervention C that he currently has access to. Cecil has a health level with intervention C of 0.9 and since the patient benefit of intervention C is 0.2, will end up at a health level of 0.7 (and an increased severity) if C is withdrawn.

Given the assumed patient benefits we get the situation summarized in Table 1. Note that the cost of A is twice the cost of B in order to keep cost-effectiveness the same between A and B.

**Table 1:** Assumptions with equal cost-effectiveness of A and B

	Current health	Cost of treatment	Effect of treatment	Cost-effectiveness of treatment
Alison	0.1	8000	0.8	10,000
Bernadette	0.1	4000	0.4	10,000
Cecil 1*	0.9	4000	0.2	20,000
Cecil 2*	0.9	4000	0.2	20,000

^*^For the Cecils the treatment is already in place and has taken them from 0.7 to 0.9

Since the costs of the interventions are now different, choosing A will require that we withdraw twice as many C-interventions as when choosing B. In the following, to handle the fact that interventions will be rationed to redistribute resources, we will assume that Cecil belongs to a patient group that contains two patients to allow such redistribution as illustrated by the new current position in Table 1. If, on the other hand, we want to keep the opportunity cost of introducing A or B the same, we will have to assume that Bernadette belongs to a group with two patients. Let us explore both options in relation to our distributive theories.

## Utilitarian perspective

In the introduction we assumed that our healthcare system is intended to promote both equity and efficiency, operationalized by balancing severity and cost-effectiveness. However, to provide a background, let us start by looking at a theory only focusing on the maximizing of benefits—utilitarianism. Starting with keeping the cost-effectiveness of interventions A and B equal (example 3a), with a utilitarian perspective we will get the situation illustrated in Table 2.

**Table 2:** Potential allocation with equal cost-effectiveness of A and B (example 3a)

	Current		Allison		Bernadette	
	Health	Cost	Health	Cost	Health	Cost
Alison	0.1		0.9	8000	0.1	
Bernadette	0.1		0.1		0.5	4000
Cecil 1	0.9	4000	0.7		0.9	4000
Cecil 2	0.9	4000	0.7		0.7	
Sum	2	8000	2.4	8000	2.2	8000

From a utilitarian perspective, intervention A is preferred since it maximizes benefits in the healthcare system. Note that this result stems from considering cost-effectiveness (optimizing patient benefit with a given budget) without independent consideration of the patient benefit.

So, let us explore what happens when we keep the opportunity cost of treating Alison and Bernadette equal (example 3b). Then we will have to assume that Bernadette belongs to a group with another patient (equal to her) as illustrated in Table 3. If so, utilitarianism will not make a difference between A and B as illustrated in Table 4.

**Table 3:** Assumptions with equal cost-effectiveness and opportunity costs of A and B

	Current health	Cost of treatment	Effect of treatment	Cost-effectiveness of treatment
Alison	0.1	8000	0.8	10,000
Bernadette 1	0.1	4000	0.4	10,000
Bernadette 2	0.1	4000	0.4	10,000
Cecil 1*	0.9	4000	0.2	20,000
Cecil 2*	0.9	4000	0.2	20,000

^*^For Cecils the treatment is already in place and has taken them from 0.7 to 0.9

**Table 4:** Potential allocations with equal cost-effectiveness and opportunity costs of A and B

	Current		Allison		Bernadette	
	Health	Cost	Health	Cost	Health	Cost
Alison	0.1		0.9	8000	0.1	
Bernadette 1	0.1		0.1		0.5	4000
Bernadette 2	0.1		0.1		0.5	4000
Cecile 1	0.9	4000	0.7		0.7	
Cecile 2	0.9	4000	0.7		0.7	
Sum	2.1	8000	2.5	8000	2.5	8000

With patient benefit higher for A and equal cost-effectiveness of A and B, opportunity costs of A and B can only be the same if the group of Bernadettes is larger than that of Alisons. In this case, the total patient benefit will be identical between the groups. A conclusion from a utilitarian perspective is that the size of the individual patient benefit does not play the role stated in our thesis (or any independent role) (Table 4). Utilitarianism will therefore not support our thesis.

## Prioritarian perspective

**Fig. 1:**
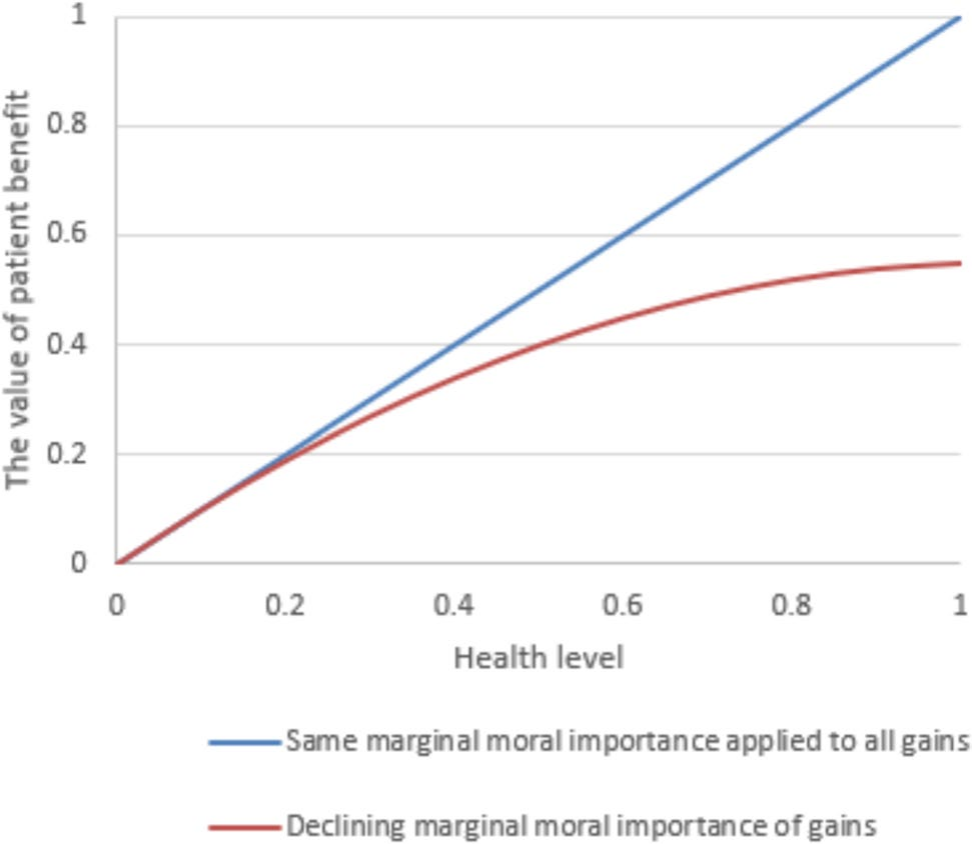
Illustration of marginal moral importance functions

Generally, prioritarianism implies that a patient benefit of a defined size has a greater moral importance if it accrues to patients who are worse off than if the same size of patient benefit accrues to patients being better off (Parfit 2000). The more exact moral importance, and its relationship to being worse off is a matter of dispute—and a discussion we will not enter into in this article (Nord & Johansen 2014). Let us, for the sake of argumentation assume the moral importance function in Table 5. In Fig. 1 the diminishing moral importance function is illustrated together with a function assigning equal weight to all gains.

**Table 5:** Assumed marginal moral importance function

Health level	Marginal moral importance of improving from previous health level	Patient benefit weighted by marginal moral importance
0		0.000
> 0–0.1	1	0.100
> 0.1–0.2	0.9	0.190
> 0.2–0.3	0.8	0.270
> 0.3–0.4	0.7	0.340
> 0.4–0.5	0.6	0.400
> 0.5–0.6	0.5	0.450
> 0.6–0.7	0.4	0.490
> 0.7–0.8	0.3	0.520
> 0.8–0.9	0.2	0.540
> 0.9–1.0	0.1	0.550

With the assumption that Bernadette belongs to a group of two patients, implying the same use of resources (opportunity cost) to treat Alison or Bernadette’s group, prioritarianism will favour B before A as illustrated in Table 6 (this corresponds to the assumptions in Tables 3 and 5). The only way to hold those two attributes constant is to allow two gains of 0.4 to be compared with one gain of 0.8.

**Table 6:** Potential allocations including marginal moral importance with equal cost-effectiveness and opportunity costs of A and B

	Current		Allison			Bernadette	
	Health	Moral importance*	Health	Moral importance		Health	Moral importance
Alison	0.1		0.9	0.44		0.1	
Bernadette 1	0.1		0.1			0.5	0.3
Bernadette 2	0.1		0.1			0.5	0.3
Cecile 1	0.9		0.7	− 0.05		0.7	− 0.05
Cecile 2	0.9		0.7	− 0.05		0.7	− 0.05
Sum	2.1		2.5	0.34	0	2.5	0.5

Here the size of the patient benefit is relevant, given the prioritarian consideration of the initial health level (severity) of the patients, which contrasts the prioritarian with the utilitarian perspective. However, in distinction to the initial intuition that prioritarianism would favour the larger individual patient benefit, the conclusion is that a smaller patient benefit might in fact be prioritized before a larger one if cost-effectiveness and opportunity costs are held constant, since it will result in a more equitable distribution. Therefore, prioritarianism does not support our thesis, rather the opposite.

## Egalitarian perspective

Our third distributive perspective is telic egalitarianism, where the equality of a distribution is one central factor to consider (Temkin 2003; Hirose 2015). In this article it is impossible to explore every version of telic egalitarianism or resulting instrument for measuring equality but let us try using the GINI coefficient to illustrate the changes of equality to the system with different distributions. If this approach indicates a similar pattern as above, we will at least have one version of telic egalitarianism according to which the initial intuition always supporting the larger patient benefit would be wrong.

As in the two previous examples we are starting with the situation where Bernadette belongs to a group of two, in distinction to Alison, to keep the opportunity cost equal (which corresponds to the examples in Tables 4 and 6) and hence affect the same number of individuals in Cecil’s group. We then have the situation illustrated in Table 7.

**Table 7:** Potential allocations including GINI coefficients with equal cost-effectiveness and opportunity costs of A and B

	Current		Allison		Bernadette	
	Health	Gini	Health	Gini	Health	Gini
Alison	0.1		0.9		0.1	
Bernadette 1	0.1		0.1		0.5	
Bernadette 2	0.1		0.1		0.5	
Cecile 1	0.9		0.7		0.7	
Cecile 2	0.9		0.7		0.7	
Sum	2.1	0.46	2.5	0.35	2.5	0.22

Interestingly enough, we see the same result from this egalitarian perspective as was seen from a prioritarian perspective; that smaller individual benefits might in fact be prioritized before larger individual benefits. In effect, at least one version of egalitarianism does not support our thesis. More generally, we could argue that how the size of the patient benefit will affect the distribution will depend on how other patients are situated in the population together with which version of equality we adhere to. Still, all different versions will imply that we sometimes should prioritize the larger individual patient benefit, and sometimes not (Temkin 1993). Hence, no version of egalitarianism would generally support our thesis.

## A last theory to explore

So far we have found no general support for the idea that the larger patient benefit should always be prioritized ceteris paribus. Before drawing any final conclusions, let us briefly look at the only distributive theory we think could potentially provide some support, sufficientarianism.

According to sufficientarianism, the way in which goods are distributed only matters up to a certain threshold (or matters significantly more up to the threshold than above it). More specifically, sufficientarianism is constituted by two theses, sometimes referred to as the positive thesis and the negative thesis (Casal 2007). The former implies that there are special moral reasons to benefit people below the threshold. The latter entails that there are no reasons, or weaker reasons, to additionally lift the people already located above the threshold further. In the original version of the theory sketched by Frankfurt (1984; 1987), sometimes called “head-count sufficientarianism” (Hirose 2015) this implies bringing as many patients as possible over this threshold. A central aspect of this theory is obviously where the threshold is set. In Table 8 we illustrate how different thresholds will impact on the role of patient benefit (with the same assumption about group size as in the later examples above).

**Table 8:** Potential allocations including sufficiency outcomes with equal cost-effectiveness and opportunity costs between A and B

	Current				Allison				Bernadette			
	Health	0.8*	0.7*	0.5*	Health	0.8*	0.7*	0.5*	Health	0.8*	0.7*	0.5*
Alison	0.1	0	0	0	0.9	1	1	1	0.1	0	0	0
Bernadette 1	0.1	0	0	0	0.1	0	0	0	0.5	0	0	1
Bernadette 2	0.1	0	0	0	0.1	0	0	0	0.5	0	0	1
Cecile 1	0.9	1	1	1	0.7	0	1	1	0.7	0	1	1
Cecile 2	0.9	1	1	1	0.7	0	1	1	0.7	0	1	1
Sum	2.1	2	2	2	2.5	1	3	3	2.5	0	2	4

^*^Sufficiency threshold (0 implies individual is below the threshold and 1 that individual is at, or above, the threshold)

In this case prioritization depends on where the threshold is set. On the other hand, also in this situation, the size of the patient benefit will not impact the decision in the same way in all possible situations (Table 8). Where we set the threshold, will affect whether a large or smaller benefit should be prioritized. Also with a fixed threshold, sometimes a large benefit should be prioritized (since it reaches the threshold), and sometime a smaller benefit (since it reaches the threshold). Hence, sufficientarianism does not generally support our thesis.

## Conclusions and implications for practice

In exploring three dominating theories of distributive justice (and a complementary theory), and given assumptions about keeping severity, cost-effectiveness, and opportunity costs constant for interventions with different patient benefits, we do not find support for the thesis that a larger patient benefit should generally be prioritized before a smaller patient benefit ceteris paribus. Does this provide unanimous, unwavering support for our thesis? Not necessarily, it still raises the question of which out of the theories of distributive justice is the most plausible one. It could of course be the case that some theory, or combination of theories, that would provide support for the thesis is that one. However, with regard to mid-level principles for priority setting, our argument seems to provide enough support against our thesis and lacking a strong argument to the contrary, a reasonable basis for not using patient benefit as an independent criterion for priority setting.

This is a relevant conclusion for priority practice and the frameworks using both patient benefit and cost-effectiveness, assuming that a larger patient benefit would always increase the priority of an intervention. Hence, for example the Swedish framework and NICE’s guidelines about prioritizing large patient benefits for highly specialized technologies appear misguided (Prop. 1996/97:60, NICE 2017). The Norwegian approach, or the EVIDEM framework (Goetghebeur & Cellier 2018), would thus be a better approach, separating cost and patient benefit (and not give an independent role to patient benefit if it is used in a cost-effectiveness assessment).

In systems where the mid-level approach used has affinities to prioritarianism, like the Swedish and Norwegian systems, our analysis, on the contrary, indicates that the moral importance of prioritizing two patients with a 0.4 patient benefit is higher than the moral importance of prioritizing a 0.8 patient benefit for one patient. Hence that we should prioritize smaller gains to more patients with severe conditions rather than large gains to smaller populations. However, the implications of formalizing such an approach in real-world priority setting must be analysed further.

## Footnotes

1 If the conditions that treatment C and D target are currently untreated, and the acceptance threshold of cost-effectiveness is €20 000, this would occur if for example the cost of C is €200 000 with an associated patient benefit of 10 and the cost of D is €100 000 with an associated patient benefit of 5.

2 We refer to patient benefit as a gain in a relevant health outcome. For the purpose of our investigation, we do not define what this health outcome is exactly or how it was estimated.

3 To exemplify: assume that the healthcare system at the margin can produce a unit of patient benefit at a cost of €20,000 and that the additional cost of a new treatment for a very severe condition is €60,000 with an incremental gain of one unit of patient benefit. Giving priority to this treatment (i.e. including it in our constrained healthcare system) implies that €60,000 must be found elsewhere in the healthcare system to fund it. This is equivalent to displacing 3 units of patient benefit elsewhere in the healthcare system while gaining 1 unit of patient benefit with the new intervention.

4 Here we assume that it is ethically acceptable to withdraw treatment from Cecil for priority reasons and thus, the equivalence thesis between withholding and withdrawing treatment is true (See for example Wilkinson & Savulescu 2014, Sandman & Liliemark 2018).

5 One way to interpret this is to claim that patient benefit is a tie-breaker, when all else is equal. This is not what we are exploring here per se. Rather, we are keeping severity and cost-effectiveness equal, to explore whether greater patient benefit should have a role. If we do not find support for our thesis, there might still be another role (not only a tie-breaker role) for patient benefit.
